# Supplementary material for: Screening and Genomic Analysis of Bacillus velezensis R12 as a Biocontrol Agent Against Fusarium oxysporum Causing Wilt in Longya Lily (Lilium brownii var. viridulum)
Source: Microorganisms. 2025 Oct 23;13(11):2430. doi: 10.3390/microorganisms13112430 (PMC12654850; doi:10.3390/microorganisms13112430)
Supplement: Supplementary file 1 [file microorganisms-13-02430-s001.zip › microorganisms-3890992-supplementary.pdf]

Supplementary Materials

Table S1. General database annotation statistics

Database	Number	100<=length<300	length>=300
nr_Annotation	3803	1770	1540
GO_Annotation	2993	1381	1384
kegg_Annotation	2171	930	1128
eggNOG_Annotation	3024	1403	1454
Pfam_Annotation	3392	1580	1505
Swissprot_Annotation	2829	1243	1392
TrEMBL_Annotation	3798	1770	1540
All_Annotated	3810	1771	1540

Note: Database: Functional database(s) used for annotation; Number: Number of annotated genes; 100 ≤ length < 300: Genes with lengths between 100 and 300 bp; length ≥ 300: Genes with lengths ≥ 300 bp.

Table S2. Prediction of secondary metabolite biosynthetic gene clusters in R12

Cluster no.	Type	Most similar known cluster		Similarity Confidence
1	NRPS	surfactin	NRPS:Type I	High
2	phosphonate	-	-	-
3	PKS-like	-	-	-
4	terpene	-	-	-
5	transAT-PKS	macrolactin H	PKS	High
6	transAT-PKS, T3PKS, NRPS	bacillaene	NRPS:Type I+PKS:Type l	High
7	NRPS, transAT-PKS, betalactone	fengycin	NRPS:Type I	High
8	terpene	-	-	-
9	T3PKS	-	-	-
10	transAT-PKS	difficidin	PKS	High
11	terpene-precursor	-	-	-
12	terpene-precursor2,NRP-metalloph ore,NRPS RiPP-like	bacilibactin	NRPS:Type l	High
13	NRPS	-	-	-
14	other	bacilysin	other:other	High
15	lanthipeptide-class-ii	mersacidin	ribosomal:RiPP:Lanthipeptide	High

Note: The symbol "–" indicates that no significant similarity to a known cluster was detected.

Supplementary.1 Isolation, Purification, and Pathogenicity Assay of Pathogenic Fungi

Pathogens were isolated using the tissue separation method. Lily bulbs (*Lilium brownii* var. *viridulum*) exhibiting distinct disease symptoms were selected as source material. The bulbs were washed, surface-sterilized by immersion in 75% ethanol, and rinsed 3-5 times with sterile water. This was followed by immersion in 2.5% sodium hypochlorite solution and another 3-5 rinses with sterile water. After air-drying, tissue fragments (approximately 0.5 cm) from the junction between diseased and healthy tissues were excised and placed on PDA medium supplemented with 100 mg/L chloramphenicol. The plates were incubated at 25 °C for 5-7 days. Emerging fungal colonies were transferred to fresh PDA medium for purification. The purified strains were subsequently assigned identification codes.

Pathogenicity was determined using a detached bulb inoculation assay. Fresh, healthy lily bulbs were washed and surface-disinfected with 75% ethanol, followed by three rinses with sterile water. Subsequently, they were treated with 1.5% sodium hypochlorite for 3 minutes and rinsed 3-5 times with sterile water. Excess moisture was removed using sterile filter paper. The bulb surfaces were wounded at seven locations using a sterile pipette tip. A 5-mm mycelial plug from an actively growing culture of the pathogen was placed onto each wound site with the mycelial side in contact with the tissue. Control bulbs were inoculated with sterile PDA agar plugs. All inoculated bulbs were maintained at 28 °C for 5-10 days. The experiment was performed in triplicate.

The pathogenic strain L1-1, purified from decayed bulbs of *Lilium brownii* var. *viridulum*, caused disease in 100% of the inoculated bulbs. As shown in Figure S1, brown necrotic lesions initially appeared at the inoculation sites, followed by central tissue decay that progressively expanded.

The morphological characteristics of pathogenic L1-1 were examined on agar plates and through microscopic observation of hyphae and conidia. L1-1 exhibited rapid growth, covering the entire Petri dish (d = 90 mm) after 5 days of cultivation on PDA medium at 28°C. The fungus developed abundant hyphae and produced pink to purple pigments during growth. The hyphae were septate, forming both microconidia and macroconidia (Figure S2).

CK

L1-1

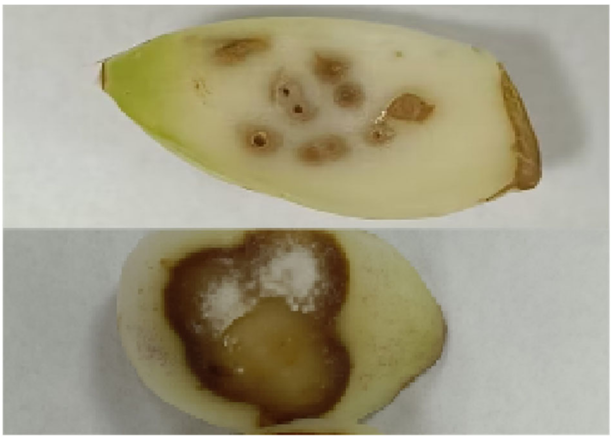

Figure S1. Pathogenicity of Strain L1-1.

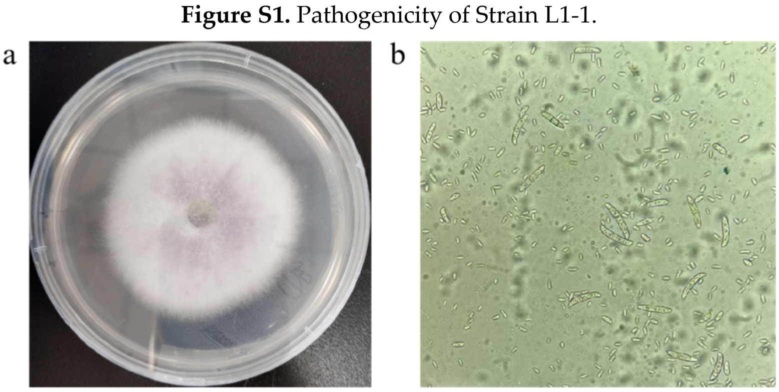

Figure S2. (a) Colony Morphology of L1-1; (b) Spore Morphology of L1-1.

Supplementary.2 Morphological and Molecular Identification of Pathogens

For morphological characterization, pathogens were inoculated onto PDA plates and cultured at 28 °C. Mycelial and spore morphology, colony color, and other cultural characteristics were observed and recorded.

Genomic DNA was extracted from the pathogens using the Solarbio Fungal DNA Extraction Kit. The extracted DNA served as the template for PCR amplification with the universal fungal primers ITS1 (5'-TCCGTAGGTGAACCTGCGG-3') and ITS4 (5'-TCCTCCGCTTATTGATATGC-3'). The PCR reaction mixture (50 μL total volume) consisted of 5 μL of 10× Ex Taq Buffer, 2 μL of Ex Taq polymerase, 2.5 μL of each primer (ITS1 and ITS4), 2 μL of DNA template, and 36 μL of ddH_2O. The amplification protocol was: initial denaturation at 95 °C for 5 min (1 cycle); followed by 30 cycles of denaturation at 95 °C for 30 s, annealing at 55 °C for 30 s, and extension at 72 °C for 1 min; with a final extension at 72 °C for 5 min before holding at 4 °C. The PCR products were purified and sequenced by Sangon Biotech (Shanghai) Co., Ltd. The obtained sequences were compared against the NCBI database, and a phylogenetic tree was constructed using MEGA11 software.

Genomic DNA was extracted from the pathogenic strain L1-1 and amplified with universal primers ITS1/ITS4 targeting the rDNA-ITS region. The sequencing results were analyzed by BLAST alignment, and a phylogenetic tree was constructed using MEGA 11.0 software. As shown in Figure S3, a 499-bp fragment was successfully amplified from strain L1. BLAST analysis revealed 99.60% sequence similarity to *Fusarium oxysporum* M0620.1030. Phylogenetic analysis demonstrated that strain L1 clustered on the same branch as *Fusarium oxysporum* M0620.1030. Based on integrated morphological and molecular identification, the pathogenic strain L1 was identified as *Fusarium oxysporum*.

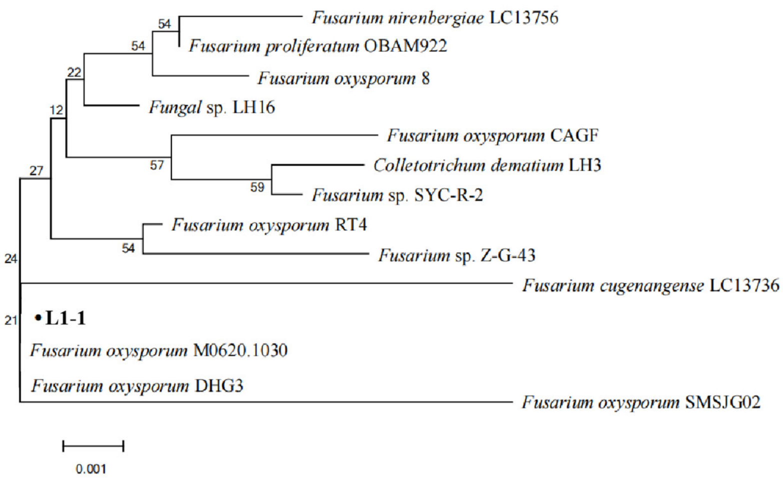

Figure S3. Phylogenetic Tree of Pathogenic Strain L1-1.
